# Spontaneous isolated dissection of the superior mesenteric artery and aneurysm formation resulting from segmental arterial mediolysis: a case report

**DOI:** 10.1186/s13000-017-0664-x

**Published:** 2017-10-16

**Authors:** Nobuhiro Akuzawa, Makoto Kurabayashi, Tsukasa Suzuki, Daisuke Yoshinari, Mitsunobu Kobayashi, Yoshifumi Tanahashi, Fujio Makita, Ryusei Saito

**Affiliations:** 1 Departments of General Medicine, National Hospital Organization Shibukawa Medical Center, 383 Shiroi, Shibukawa, Gunma 377-0280 Japan; 2 Surgery, National Hospital Organization Shibukawa Medical Center, 383 Shiroi, Shibukawa, Gunma 377-0280 Japan; 3 Pathology, National Hospital Organization Shibukawa Medical Center, 383 Shiroi, Shibukawa, Gunma 377-0280 Japan; 4 Respiratory Medicine, National Hospital Organization Shibukawa Medical Center, 383 Shiroi, Shibukawa, Gunma 377-0280 Japan

**Keywords:** Aneurysm formation, Computed tomography, Dissection, Segmental arterial mediolysis, Superior mesenteric artery

## Abstract

**Background:**

Spontaneous isolated dissection of the superior mesenteric artery (SMA) can lead to bowel ischemia, aneurysm rupture, or even death. Studies have suggested that mechanical or hemodynamic stress on the vascular wall of the SMA may be a contributor, but its pathogenesis is unclear.

**Case presentation:**

A 57-year-old Japanese man with a history of untreated hypertension and hyperuricemia was admitted to our hospital with the sudden onset of severe epigastric pain. Laboratory findings showed elevated white blood cell count and C-reactive protein, and contrast-enhanced computed tomography (CT) of the abdomen demonstrated arterial dissection with luminal stenosis and aneurysm formation at the distal portion of the SMA after the branching of the jejunal artery, and intravenous nicardipine was administered. The patient’s epigastric pain resolved spontaneously but recurred on day 6 of his hospital stay. Contrast-enhanced abdominal CT revealed an enlarged aneurysm with wall thinning. Because of the risk of aneurysm rupture, the decision was made to perform aneurysmectomy and bowel resection on day 6. Histologic examinations revealed two separate dissecting lesions: one latent and the other resulting in aneurysm formation. Both lesions showed characteristics of segmental arterial mediolysis (SAM) with lack of arterial media, absence of internal and external elastic laminae and intimal proliferation.

**Conclusions:**

Histologic findings in the present case suggest that mechanical or hemodynamic stress on the vascular wall and SAM-related vascular vulnerability may concomitantly contribute to the onset of isolated SMA dissection.

## Background

Spontaneous isolated dissection of the superior mesenteric artery (SMA) is considered rare, but the number of reports of SMA dissection is increasing, reflecting the widespread use of contrast-enhanced computed tomography (CT) [1, 2]. The two major therapeutic strategies for SMA dissection are i) endovascular treatment or surgical revascularization, and ii) conservative treatment with anticoagulation [1, 3]. In patients with isolated SMA dissection followed by aneurysm progression, surgical repair or endovascular treatment may be warranted [3]. The predominance of relatively young, male patients and cigarette smokers among patients with spontaneous SMA dissection has been reported [4, 5]. However, the causes and risk factors related to the onset of spontaneous SMA dissection are still unclear.

A plausible cause of isolated SMA dissection is involvement with segmental arterial mediolysis (SAM), which is characterized histologically by lysis of the outer media of the arterial wall leading to vascular dissection and subsequent aneurysm formation [6]. SAM is also defined as a nonatherosclerotic, nonhereditary vasculopathy without an inflammatory component [6].

We present a case involving a male patient with isolated spontaneous dissection of a trunk of the SMA with subsequent aneurysm formation on hospital day 6. He underwent aneurysmectomy and partial small-bowel resection on the same day, and a portion of this resected aneurysm was sent for pathologic examination. Dissection was evident on gross inspection, and histologic examination revealed characteristics consistent with SAM.

## Case presentation

A 57-year-old Japanese man presented to the emergency department with sudden onset of severe epigastric pain. His medical history included untreated hypertension and hyperuricemia for 3 years; Family history was unremarkable. He was a nonsmoker and did not consume alcohol. He was admitted to the hospital for further treatment.

On admission, his height was 165 cm and weight, which had been unchanged over the past 10 years, was 49 kg. His body temperature was 36.5 °C, and blood pressure was 168/94 mmHg. His heart rate was 90 beats/min and regular. With the exception of tenderness on palpation over the epigastrium, his physical examination was unremarkable. Blood tests showed elevated white blood cell count (WBC, 16,200/mm^3^ [normal range, 3300–8600/mm^3^]) as well as elevated levels of lactate dehydrogenase (246 U/L [normal range, 124–222 U/L]), uric acid (529 μmol/L [normal range, 230–420 μmol/L), and C-reactive protein (CRP, 39.8 mg/L [normal, <1.5 mg/L]). Hemoglobin concentration was normal (141 g/L; normal, 137–168 g/L). Serum concentrations of lactic acid, electrolytes, and bicarbonate were normal, as was his anion gap. An abdominal X-ray was unremarkable.

**Fig. 1:**
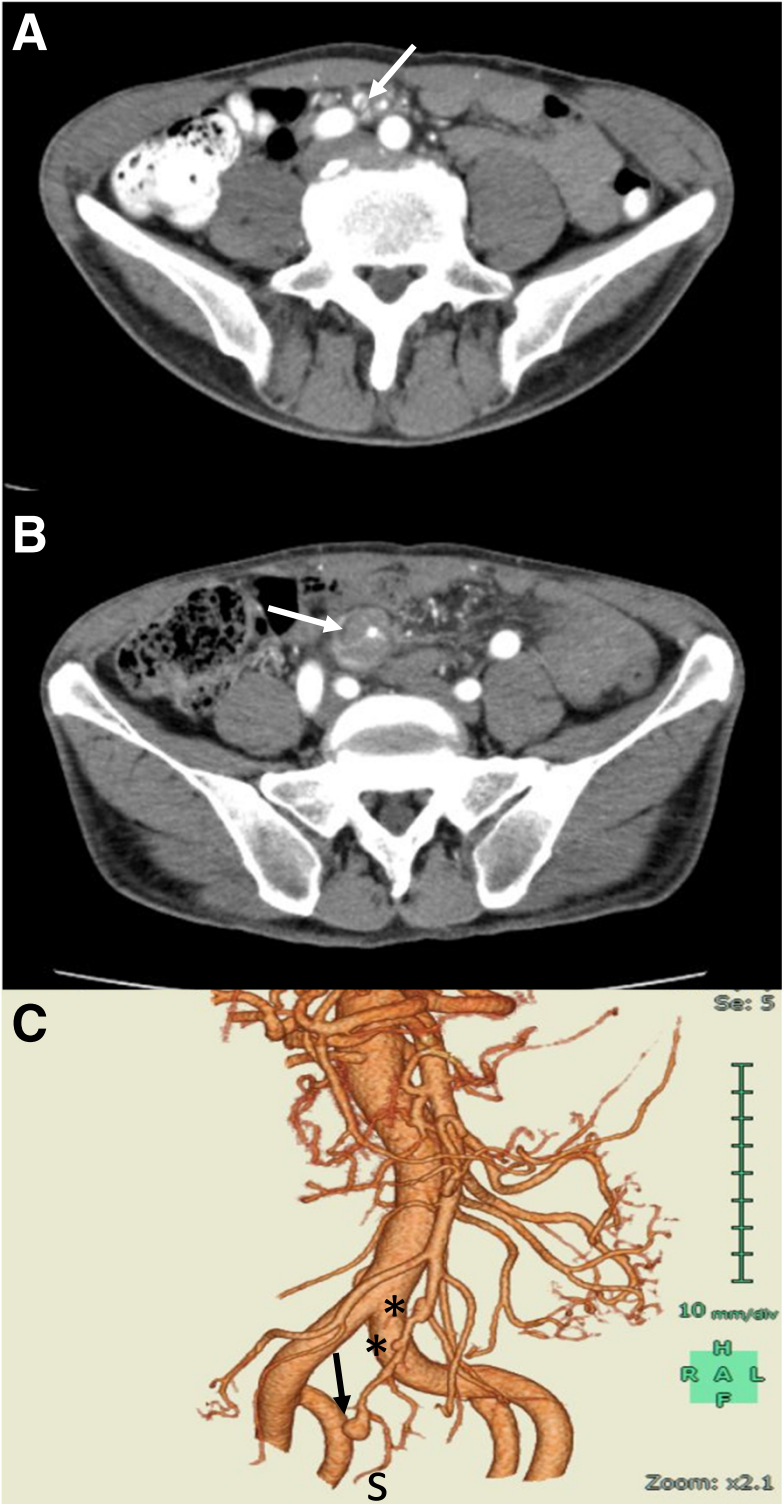
Contrast-enhanced CT images on admission. **a** *Superior* mesenteric artery (white arrow) showing luminal stenosis and concentric intramural hematoma without extravasation. **b** A small intimal flap (white arrow) can be seen inside the SMA just proximal to the intramural hematoma. **c** CT angiograph showing two lesions (asterisks) with segmental dilatation proximal to the SMA aneurysm. A blind-sac aneurysm (black arrow) can also be seen. At this point, SMA blood flow distal (S) to the aneurysm is preserved

Contrast-enhanced abdominal CT demonstrated an intimal flap inside the SMA at the proximal portion of the aneurysm (Fig. 1a), suggesting that aneurysm formation might have ensued from spontaneous SMA dissection. Luminal stenosis of the SMA, with concentric intramural hematoma without extravasation, and aneurysm formation at the distal portion of the SMA were also observed (Fig. 1b). Notably, CT angiography demonstrated two lesions showing segmental dilatation proximal to the SMA aneurysm (Fig. 1c). Because of the risk of SMA aneurysm rupture, antihypertensive therapy was started; specifically, continuous intravenous infusion of nicardipine (2 μg/kg/min) was begun soon after obtaining the CT, and the patient’s blood pressure decreased to about 110/60 mmHg. Although there was narrowing of the SMA, especially in the proximal portion of the aneurysm, anticoagulation therapy was not instituted.

**Fig. 2:**
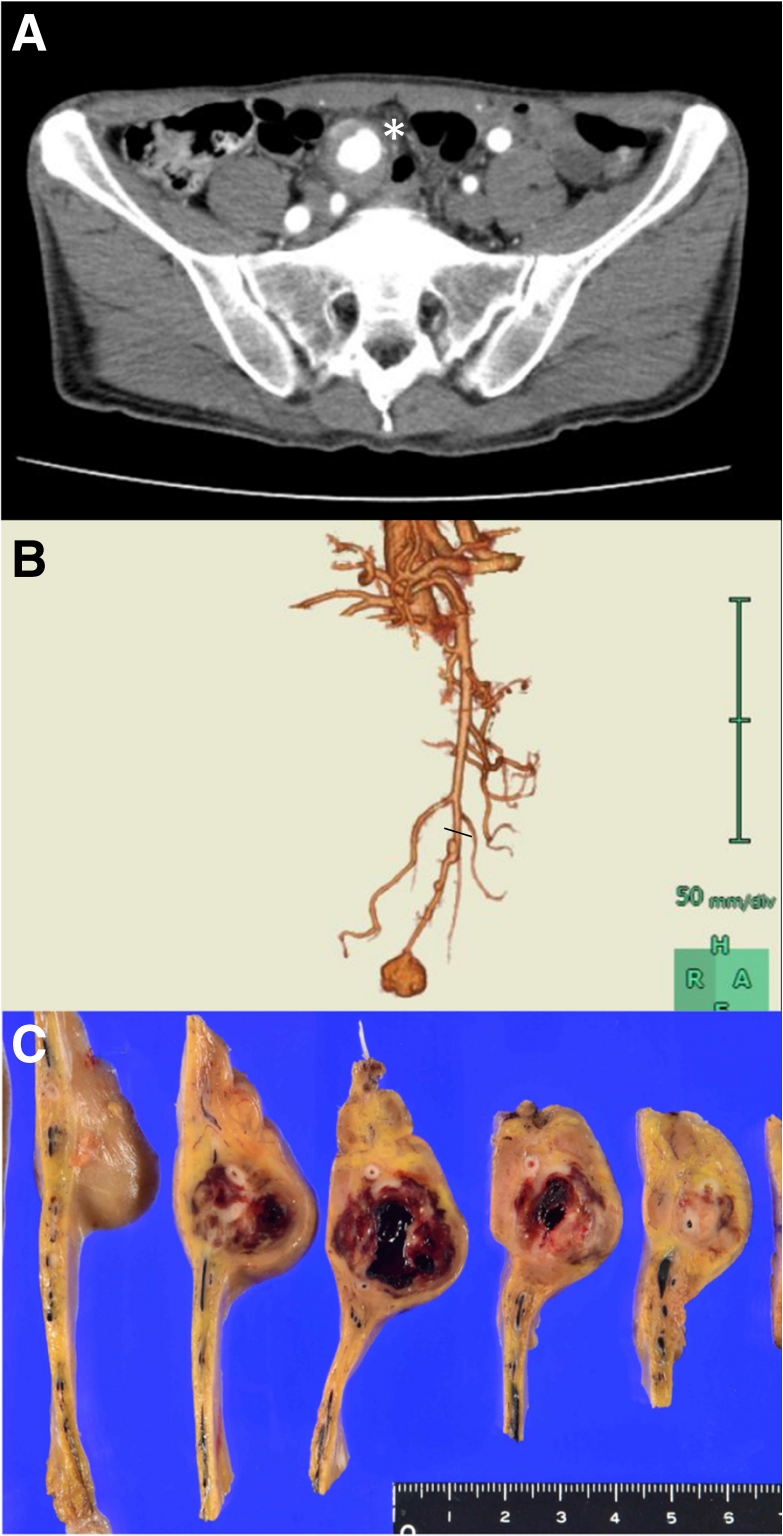
Preoperative contrast-enhanced CT images on day 6 and photograph of resected specimen. **a** Aneurysm (white arrow) became larger in size, and its wall thickness became thinner, compared with the CT findings on admission. **b** CT angiograph revealed disappearance of SMA blood flow distal to the aneurysm that had been detected on CT angiograph on admission (Fig. 1c), strongly suggesting obstruction of the SMA distal to the aneurysm. Black line indicates the position of the SMA resection stump during bowel resection. **c** Gross inspection of the resected specimen reveals formation of a pseudoaneurysm in the mesentery

On hospital day 2, the patient’s epigastric pain persisted. Contrast-enhanced abdominal CT was again performed, but no obvious changes were seen compared with the CT findings on admission. Thereafter, his epigastric pain gradually resolved. By hospital day 4, the patient’s WBC count had normalized to 5400/mm^3^ and serum CRP had decreased to 15.4 mg/L. The patient’s epigastric pain had completely resolved by hospital day 5 but recurred on day 6. Contrast-enhanced abdominal CT on the same day revealed a notable increase in the size of the SMA aneurysm to 31 × 29 mm, from 23 × 24 mm on admission, and a decrease in the aneurysm’s wall thickness in some areas (Fig. 2a). CT angiography strongly suggested occlusion of the distal SMA because SMA blood flow distal to the aneurysm, which had been detected on CT angiograph on admission, disappeared (Fig. 2b). WBC count and serum CRP level were also elevated to 8700/mm^3^ and 37.6 mg/L, respectively. Because of the risk of aneurysm rupture and ischemic bowel necrosis, the decision was made to perform surgical resection of the SMA aneurysm and partial distal ileum; the length of the resected ileum was 43 cm. This was performed on day 6. During the bowel resection procedure, the SMA was divided at a point showing segmental vasodilation, just proximal to the lesion (Fig. 2b). Gross inspection revealed no obvious bowel necrosis, but there was evidence of the formation of a pseudoaneurysm inside the mesentery (Fig. 2c). The SMA distal to the pseudoaneurysm was occluded by thrombus.

**Fig. 3:**
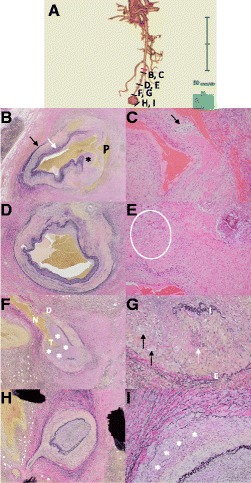
Histopathologic findings of resected specimen. **a** CT angiograph of the SMA on Day 6 indicating the origin of each section; letters A through I correspond to Fig. 3a through i (black lines). Red line indicates the position of the SMA resection stump. **b** Low-power view (LPV) (×100) with Elastica van Gieson (EVG) staining of the SMA adjacent to the proximal resection stump. Internal (white arrow) and external (black arrow) elastic laminae as well as arterial media have partially disappeared and there is prominent intimal proliferation (asterisk). On CT angiography, this region showed only mild vasodilatation but an entry point of latent dissection was seen. “P” indicates pseudolumen filled mainly with fibrin. **c** High-power view (HPV) (×400) with hematoxylin and eosin (HE) staining shows vacuolization (black arrow) and marked decrease in the number of vascular smooth muscle cells (SMCs). **d** LPV (×100) with EVG staining of the area slightly distal to the lesion shows latent dissection with preserved internal elastic lamina and eccentric intimal proliferation. **e** HPV (×400) with HE staining also shows a vacuolization-rich area (ellipse) in the arterial media and disturbed arrangement of medial SMCs. **f** LPV (×100) of the SMA with EVG staining of the area adjacent to the pseudoaneurysm neck (N) showing remarkable stenosis of the true lumen (T) due to intimal proliferation (asterisks). A small dissection (D) is also observed, but wall rupture and resultant aneurysm formation are predominant. Arterial wall adjacent to aneurysm neck lacks medial SMCs. **g** HPV (×400) with EVG staining of the SMA adjacent to the neck of the pseudoaneurysm. Arterial media between internal (I) and external (E) elastic laminae shows focal vacuolization (black arrows) and degeneration of vascular SMCs (white arrow). **h** LPV (×100) with EVG staining of the SMA distal to the pseudoaneurysm. Both internal and elastic laminae are preserved and intimal proliferation is unremarkable. Arterial lumen is occluded with thrombus. **i** HPV (× 400) of the distal SMA with EVG staining. There is not a great deal of vacuolization, but degeneration and disarrangement of the outer media (asterisks) are visible

Histopathologic examination of specimens of the resected SMA focused on the four regions shown in Fig. 3a. On microscopic examination, there was latent dissection inside the lesion, with vasodilatation adjacent to the proximal resection stump, and the pseudolumen of the dissection was filled with fibrin (Fig. 3b). In addition, the internal and external elastic membranes of the SMA had partially disappeared, and the lesion was seen to have medial vacuolization and lysis without infiltration of inflammatory cells (Fig. 3c). In an area slightly distal to this lesion, the internal elastic membrane was preserved (Fig. 3d), but partial vacuolization and lysis in the arterial media and eccentric intimal proliferation remained (Fig. 3e). At the neck of the SMA aneurysm, dissection of the arterial media was evident, and both medial vacuolization and partial disappearance of internal elastic membrane were again observed. Notably, remarkable stenosis of the true lumen due to intimal proliferation was evident (Fig. 3f, g). The arterial wall at the distal aspect of the SMA aneurysm showed a normal structure of internal elastic membrane and notably less vacuolization in the arterial media (Fig. 3h, i).

Postoperatively, the patient was in good condition. Intravenous administration of nicardipine was discontinued on hospital day 12 (postoperative day 6), and oral amlodipine (5 mg/day), valsartan (80 mg/day) and allopurinol (100 mg/day) were started on hospital day 13 (postoperative day 7). He was discharged from our hospital on hospital day 14 (postoperative day 8). The patient has now been followed for 6 months and has had no recurrence of SMA dissection or associated aneurysm formation.

## Discussion and conclusions

The reported incidence of isolated SMA dissection is 0.06% and the SMA is the second most frequent peripheral artery after the internal carotid artery to be affected by spontaneous dissection [7, 8]. Unlike in aortic dissection, about 30–40% of patients with isolated SMA present with hypertension [2]. SMA dissection commonly begins 1.5–3.0 cm from the orifice of the SMA, corresponding to the retropancreatic portion, where the SMA is fixed in position. In contrast, the distal portion of the SMA is relatively mobile, suggesting that the curvature of this boundary zone is susceptible to mechanical stress [9]. A recent computer-simulation model also suggested that the anterior wall of the curvature is constantly affected by hemodynamic stress [8]. Accordingly, mechanical or hemodynamic stress on the vascular wall of the SMA may be the leading cause of isolated SMA dissection. However, in the present case, the lesions of isolated SMA dissection were found in separate portions of the SMA. In addition, it should be noted that these lesions were not connected to each other and that the locations of these lesions were quite separate from SMA orifice. One lesion was presumably older than the other, and the other lesion had led to aneurysm formation. Histopathologic findings revealed non-inflammatory vacuolization in the arterial media and no obvious signs of bacterial infection around the aneurysm. Thus, these findings strongly suggest that SAM might have contributed to the onset of both SMA dissection and aneurysm formation in this case.

SAM has been reported in late middle-aged and elderly populations, with no difference between the sexes [6]. SAM most often affects abdominal visceral arteries and can result in arterial dissection, hemorrhage, or ischemia that usually manifests as severe abdominal pain, but it can also affect the renal, coronary, and intracranial arteries [6]. When acute SAM-related symptoms intensify, resection of affected bowel and/or resection of aneurysm(s) can be performed, but the long-term prognosis of SAM is unclear. However, it should be noted that complete or partial resolution of SAM lesions may occur spontaneously [6].

Pathologic classification includes four diagnostic lesions: i) mediolysis, ii) arterial gaps, iii) separation, and iv) reparative fibrosis [6]. Mediolysis is characterized by vacuolization and lysis of the outer arterial media leading to subsequent formation of arterial gaps, loss of the external elastic lamina, and separation of media from adventitia. Consequent onset of dissection at the arterial gap can result in massive hemorrhage or aneurysm formation. Tissue repair characterized by granulation tissue and fibrosis also commences at the dissection lesion, and this results in vessel remodeling and restoration of the arterial wall [6].

Recently, CT and CT angiography have been widely used to diagnose SAM because of the difficulty obtaining pathology specimens from SAM-affected vessels [6]. Typical findings related to SAM on CT or CT angiography are fusiform aneurysm, stenosis, dissection, and occlusion [6, 10]. A pattern of aneurysms and stenoses in series, or a *string-of-beads* appearance, is characteristic. In addition, dissection of the peripheral arteries is radiographically one of the hallmark lesions related to SAM [6]. With regard to the pathogenesis of SAM, repeated vasoconstrictive stimuli may be associated with the development of SAM [6]. Slavin et al. [11] demonstrated in canine models that iatrogenic or accidental exposure to α1 or β2 adrenergic receptor agonists leading to norepinephrine release from the peripheral nervous system may be associated with the onset of SAM. The predominance of cigarette smokers among patients with spontaneous SMA dissection suggests that nicotine-induced catecholamine release may be related to the onset or progression of SAM [4, 5, 12].

In the present case, two dissection lesions were observed. One lesion was probably latent, and the other resulted in symptomatic aneurysm formation. Notably, both lesions showed patchy, isolated destruction of the arterial media involving both the internal and external elastic laminae. Moreover, in areas adjacent to destruction of the media, intimal proliferation with luminal stenosis was observed. In both lesions, the entry point of the dissection corresponded to the region where the intima and adventitia were immediately adjacent to each other because of the absence of arterial media; this suggests that structural vulnerability from the lack of media and loss of elastic laminae may be strongly associated with the onset of dissection. Indeed, the ulcer-like projection at the SMA dissection lesion, seen on contrast-enhanced CT, may indicate a high risk of aneurysm formation and reflect this structural vulnerability [13]. Interestingly, in a case of ischemic colitis resulting from SAM-induced occlusion of the left colic artery, Baker-LePain et al. [14] reported histologic findings that were quite similar to those in the present case, including intimal proliferation and lack of media. These findings may indicate that similar SAM lesions can cause both arterial stenosis and dissection. In the present case, luminal stenosis due to intimal proliferation was most notable at the dissection entry point. This stenosis may also affect mechanical or hemodynamic stress on the vascular wall leading not only to the onset of dissection, but also to subsequent aneurysm formation because of the turbulent flow generated by a steep pressure drop across a stenotic lesion [15]. Moreover, a loss of elasticity of SAM lesions resulting from disarrangement of vascular smooth muscle cells or elastic laminae can increase the risk of dissection or aneurysm rupture [16]. In addition, findings of our case also suggest that visceral ischemia due to SMA dissection may be caused by either compression of the true lumen by an enlarged false lumen or SAM-related preexisting luminal stenosis at a dissection entry point.

The present case indicates that stenotic lesion may become an entry point of dissection leading to aneurysm formation. To clarify this point, further analysis of histopathology and imaging, such as CT or CT angiographic images, should be conducted in SAM patients.

In conclusion, we have presented a case of a SMA aneurysm resulting from SAM-related SMA dissection. Because of the risk of rupture of the aneurysm, the patient underwent surgical resection of the SMA aneurysm and partial small-bowel resection. Histologic examination revealed two separate lesions with SAM-related dissection. Both lesions showed a lack of media and proliferation of intima, and one lesion showed luminal stenosis that resulted in aneurysm formation. These findings suggest that that mechanical or hemodynamic stress on the vascular wall and SAM-related vascular vulnerability may concomitantly contribute to the onset of isolated SMA dissection.

## Abbreviations

CT: Computed tomography
EVG: Elastica van Gieson
HE: Hematoxylin and eosin
HPV: High-power view
LPV: Low-power view
SAM: Segmental arterial mediolysis
SMA: Superior mesenteric artery
SMC: Smooth muscle cell
WBC: White blood cell
